# Artificial Intelligence Meets Dementia Care: Co‐Production for Accessibility and Inclusivity in the LUMEN Project

**DOI:** 10.1002/alz70861_108467

**Published:** 2025-12-23

**Authors:** Judith Rose Harrison, Mohamed Elsharif, John‐Paul Taylor

**Affiliations:** ^1^ Translational and Clinical Research Institute, Faculty of Medical Sciences, Newcastle University, Newcastle upon Tyne, England UK; ^2^ Cumbria, Northumberland, Tyne and Wear (CNTW) NHS Foundation Trust, Newcastle UK; ^3^ Translational and Clinical Research Institute, Newcastle University, Newcastle upon Tyne UK

## Abstract

**Background:**

Artificial intelligence (AI) has transformative potential in dementia care. However, for such tools to be effective, they must be designed to meet the diverse needs of patients, carers, and healthcare professionals. The LUMEN project (Large Language Model for Understanding and Monitoring Elderly Neurocognition) is developing an AI‐assisted tool for dementia assessment, which uses a Large Language Model to take structured collateral histories from a patient’s relatives or carers. Co‐production with stakeholders is integral to ensuring LUMEN is not only clinically effective but also user‐friendly and culturally relevant across different user groups.

**Method:**

A series of co‐production workshops have been conducted with patients, carers, and healthcare professionals. Participants have been recruited from diverse cultural, linguistic, and digital backgrounds, with strategic partnerships formed with community organisations, including those from underserved communities. These workshops, currently in progress, focus on evaluating LUMEN’s interface, language clarity, and cultural appropriateness. Participants engage with the LUMEN prototype, providing feedback on language, interface usability, and overall user experience. Using a ‘Think Aloud’ methodology, participants articulate their immediate thoughts while interacting with the tool, allowing facilitators to capture valuable data on usability and engagement. Feedback is audio recorded, transcribed and systematically analysed using thematic analysis, identifying key themes and patterns that highlight challenges related to language, interface design, and cultural sensitivity.

**Result:**

Although the workshops are ongoing, preliminary analysis has identified key areas for improvement, including interface complexity and terminology usage. Participants with lower digital literacy may experience difficulties navigating the tool and understanding some of its more technical terms. Thematic analysis of the workshop transcripts will reveal deeper insights into how specific design features impact usability, with a particular focus on the cultural appropriateness of language and accessibility for users with varying levels of technical expertise.

**Conclusion:**

The LUMEN project highlights the crucial role of co‐producing artificial intelligence tools in healthcare. By integrating feedback from diverse stakeholders and community partners, LUMEN is being refined to ensure it is clinically effective, culturally sensitive, and accessible to all. This approach will not only improve dementia care but also pave the way for more inclusive, user‐friendly AI tools in healthcare.

